# The health diplomacy spiral: a conceptual framework linking health system integration and public health diplomacy

**DOI:** 10.3389/phrs.2026.1609977

**Published:** 2026-08-25

**Authors:** Aurimas Peckauskas, Arunas Dulkys, Olegas Niaksu, Ramune Kalediene

**Affiliations:** 1 Department of Health Management, Faculty of Public Health, Lithuanian University of Health Sciences, Kaunas, Lithuania; 2 Department of Anesthesiology, Faculty of Medicine, Lithuanian University of Health Sciences, Kaunas, Lithuania; 3 Department of Quantitative Methods and Modelling, Faculty of Economics and Business Administration, Vilnius University, Vilnius, Lithuania; 4 Centre for Digital Medicine, Faculty of Medicine, Vilnius University, Vilnius, Lithuania

**Keywords:** data governance, digital health, global health governance, health system integration, public health diplomacy

## Abstract

**Objectives:**

To examine the interrelations between health system integration, digital transformation and public health diplomacy and to conceptualise how systemic threats, interdependence, and governance constraints shape cross-border health coordination.

**Methods:**

A structured integrative literature review was conducted in PubMed (2015–2025), covering two domains: health system transformation and health diplomacy. Studies were selected through multi-stage screening. Thematic and conceptual synthesis was used to identify cross-domain patterns.

**Results:**

The review supported a conceptual synthesis linking systemic pressures, institutional reforms, integration processes, and cross-border coordination. Although these relationships were rarely examined directly in the included studies, cross-domain interpretation suggested convergence through interdependence driven by integration and digitalisation, alongside governance constraints that limit coordination. These conditions may create the structural basis for public health diplomacy as a practice-oriented response to transnational health risks.

**Conclusion:**

This study proposes the Health Diplomacy Spiral, a conceptual framework for examining how health system integration and digital transformation may generate interdependence and governance complexity. The framework offers an analytical lens for future empirical research on health system transformation, data governance, and international coordination.

## Introduction

Contemporary health systems are shaped by systemic threats—pandemics, geopolitical instability and hybrid risks - which elevate coordination demands beyond national capacities. In response, reforms aimed at resilience and efficiency drive health system integration, embedding digital health infostructures that generate interoperable data environments [1–5]. While these developments may enhance coordination, they also contribute to interdependence constrained by data governance, sovereignty, and regulatory fragmentation. Under such conditions, cross-border cooperation may become necessary yet contested, creating conditions in which new forms of health diplomacy centred on negotiation and coordination gain importance. These dynamics suggest a shift in diplomacy from traditional state-centric processes towards system-level interactions grounded in health system capacities and governance structures [6, 7].

Against this backdrop, public health diplomacy has emerged as a critical interface between health system governance and international cooperation. While global health diplomacy is commonly associated with negotiation and governance processes shaping the global policy environment for health [6, 7], public health diplomacy is used here as an operational lens focused on public health capacities, system resilience and societal engagement [8, 9]. The distinction between these concepts is not absolute, as both may involve state and non-state actors.

Health systems face pressure to deliver accessible, high-quality, and cost-effective care amid ageing populations and rising multimorbidity [10, 11]. These pressures have accelerated the shift towards value-based healthcare, prioritising outcomes, efficiency, and coordination over service volume [12–14]. Policymakers prioritise health system integration as a central reform strategy [13]. In this manuscript, integration is understood as a broader reform orientation through which health systems seek to strengthen coordination capacity, improve resilience, and support system-wide performance. Integration creates structural conditions for coordination capacity and interdependence across health systems [15].

Integrated care refers to coordination of services across providers and sectors to improve quality and efficiency. A distinction is made between vertical integration, linking different levels of care, and horizontal integration, connecting providers at similar levels [15, 16]. Contemporary reforms combine both approaches to address complex population health needs, improving system performance while expanding coordination capacity ([17]). This provides the rationale for focusing on integration as the domestic reform pathway through which broader questions of digital transformation, interdependence, and public health diplomacy become relevant.

Examples from England, Singapore, the Netherlands, the United States, and Slovenia illustrate this broader reform direction, with integration-oriented reforms combining community-based and digitally enabled care, regional and population health strategies, value-based financing, and strengthened coordination across levels of care [17–22]. Comparative evidence across OECD countries positions health systems along a continuum between integrated and fragmented models of care provision [23]. More integrated systems rely on stronger organisational alignment and gatekeeping. Rising chronic disease and multimorbidity have accelerated the adoption of integrated, patient-centred care models [11], indicating that integration is a systemic transformation expanding coordination capacity and embedding data-driven interdependencies within and across health systems.

Beyond efficiency and quality, integration is presented as critical for health system resilience, particularly amid systemic shocks such as COVID-19 and geopolitical tensions [3–5]. This is especially relevant for countries on the eastern border of NATO, where proximity to active conflict zones intensifies pressure to ensure continuity of care, crisis readiness and adaptability to hybrid threats. In such contexts, resilience may increasingly depend on coordination beyond national systems, reinforcing cross-border interdependence in managing health risks.

Crisis preparedness may also increase the importance of health data portability and digitalisation. As digital transformation advances, integrated systems can generate interoperable data environments that support cross-border collaboration and policy learning [1, 24]. However, differences in legislation and data governance can constrain cross-border data use, suggesting that interdependence remains shaped by national legal and regulatory limits. These dynamics suggest that integrated healthcare systems may extend beyond organisational reform, creating institutional, coordination and data infrastructures that operate across national boundaries. Existing conceptual frameworks provide important but partial foundations: integrated care frameworks focus on coordination across providers and sectors [16], global health diplomacy frameworks emphasise negotiation and governance in the global policy environment [6, 7], and digital health frameworks address interoperability and data governance [1, 24]. However, these strands are usually examined separately and do not fully explain how domestic health system integration and digital transformation may generate cross-border coordination needs relevant to public health diplomacy. Despite this, the relationship between health system integration and public health diplomacy remains underexplored. This study addresses this gap through a structured literature review and development of the Health Diplomacy Spiral framework.

## Methods

This study used a structured integrative literature review to examine the relationship between health system integration and public health diplomacy and to support conceptual framework development at the intersection of these two areas. This approach enabled transparent identification, selection, and synthesis of literature across multidisciplinary domains, which provided a methodological foundation for developing a novel framework and identifying directions for future research.

### Literature search strategy

A structured search was conducted in PubMed for publications from January 2015 to December 2025, capturing developments in health system transformation, governance, and diplomacy. PubMed was selected as the primary database due to its comprehensive coverage of peer-reviewed biomedical and public health literature, including health systems, digital health, integrated care, and health diplomacy. In addition, PubMed’s indexing of multidisciplinary journals and policy-relevant research was considered sufficient to capture the core evidence base for this review, while maintaining a focused and reproducible search strategy.

The search included two complementary domains. The first focused on health system transformation, including integrated healthcare, digital health transformation, value-based healthcare, and health system resilience and preparedness. The second focused on health diplomacy and global health governance, reflecting the evolving terminology in this field. Search terms combined MeSH and free-text keywords, with Boolean operators used to combine related terms within and across domains. The complete PubMed search strategy, including search strings and Boolean combinations, is provided in Supplementary Material 2.

### Eligibility criteria and study selection

English-language studies published between 2015 and 2025 were included if they addressed system-level governance, policy, reform, or international coordination in integrated care, digital health, value-based healthcare, resilience, health diplomacy, or global health governance. Priority was given to reviews, policy analyses, frameworks, consensus reports, and guidelines, while clinical, patient-level, single-provider, and purely technical studies were excluded. Records were screened by date, language, publication type, title, and abstract against predefined criteria.

A structured scoring framework was applied during abstract screening. Studies were evaluated for system-level relevance, alignment with core review domains, contribution to the conceptual chain, analytical or conceptual value, and relevance for manuscript synthesis. Each criterion was scored from 0 to 1 point. One additional point was awarded for publications from 2020 to 2025, giving maximum possible score of six points. Studies meeting the predefined threshold of ≥4 points were retained for full-text review. The full scoring rubric, threshold explanation, and examples of study evaluation are provided in Supplementary Material 2.

### Data analysis

Selected studies were analysed using thematic and conceptual synthesis across four domains: health system integration, digital transformation, value-based healthcare, and health system resilience. The synthesis identified recurring patterns, functional relationships between transformation processes, and links between domestic reforms and cross-border coordination. Themes were first grouped within these domains and then compared across domains to identify relationships that recurred in different parts of the literature. Themes were prioritised when they helped explain transitions between systemic pressures, reform responses, integration processes, digital transformation, governance constraints, and cross-border coordination. Rather than aggregating findings quantitatively, the analysis provided a conceptual understanding of how these domains interact and how their interaction may generate cross-border coordination dynamics. The findings were then organised into a sequential analytical structure that informed development of the Health Diplomacy Spiral framework.

## Results

### Study selection

**FIGURE 1:**
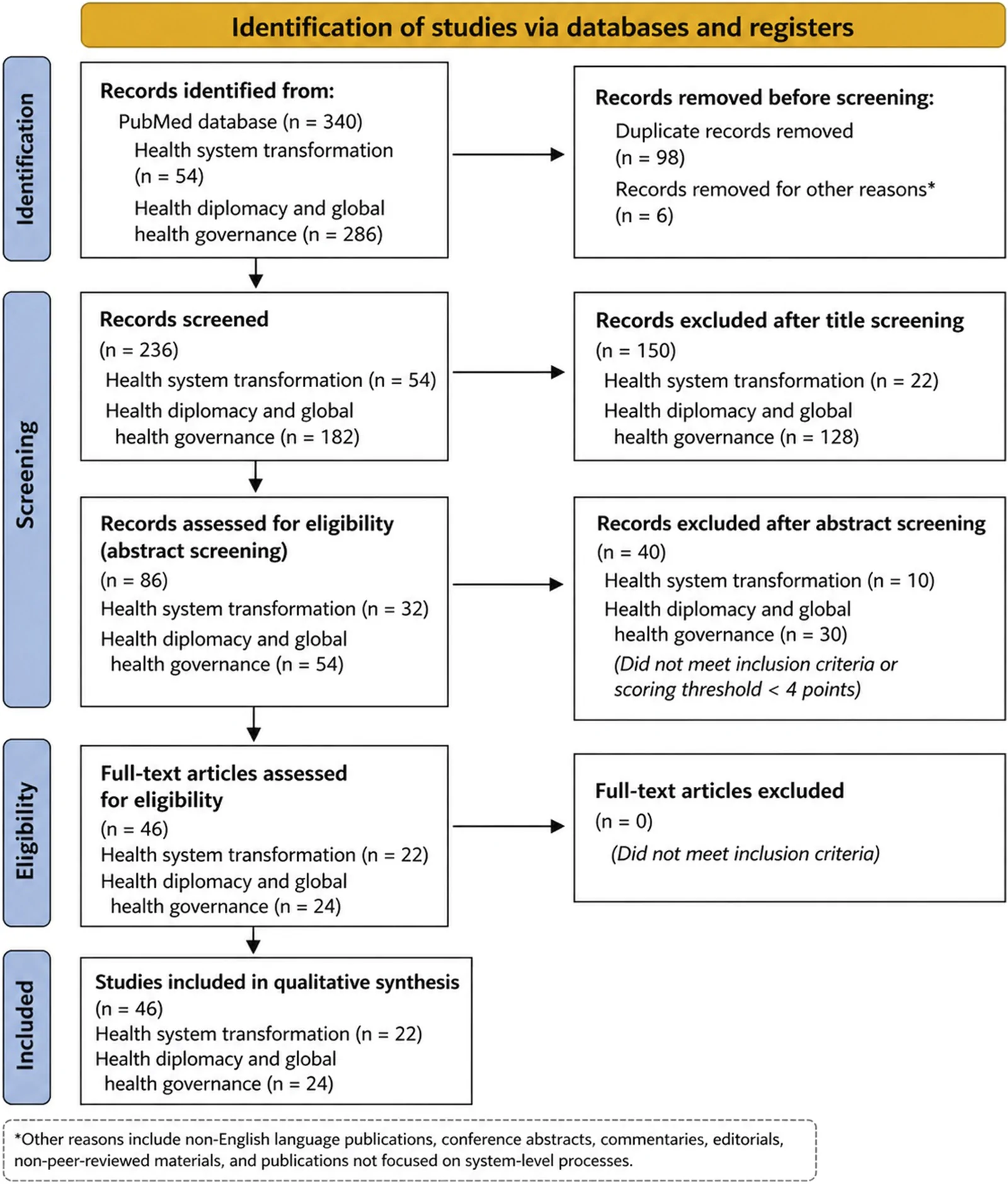
Flow diagram of the study selection process. Source Adapted from Page MJ et al. (British Medical Journal 2021; 372n71) structured integrative literature review, Global 2015–2025.

The literature search identified 54 studies in the health system transformation domain and 286 in the health diplomacy and global health governance domain. Following eligibility screening, these were reduced to 32 and 54 studies, respectively. After abstract screening using the predefined scoring threshold (≥4 points), the sample was reduced to 46 studies: 22 on health system transformation and 24 on health diplomacy and global health governance (Figure 1).

### Characteristics of included studies

The included studies consisted mainly of systematic and scoping reviews, policy analyses, and conceptual frameworks. Studies in the health system transformation domain addressed integrated care, digital health infrastructure, value-based financing, and resilience. Studies in the health diplomacy and global health governance domain focused on international cooperation, governance mechanisms, crisis coordination, and emerging concepts of public health diplomacy. Across both domains, few studies explicitly examined the relationship between health system transformation and public health diplomacy, supporting the need for conceptual synthesis. Detailed characteristics are provided in Supplementary Material 1.

### Conceptual synthesis

Although the reviewed literature does not explicitly conceptualise the relationship between health system integration and public health diplomacy, consistent patterns emerge across domains. These patterns indicate a sequential and interdependent dynamic linking systemic pressures, institutional reforms, integration processes, and cross-border coordination. Based on this synthesis, the findings are organised into a conceptual sequence informing the development of the Health Diplomacy Spiral framework.

#### Systemic threats and the reconfiguration of health system priorities

The literature indicates that contemporary health systems operate in an environment increasingly shaped by systemic threats, including pandemics, geopolitical instability, and hybrid risks. These threats disrupt service delivery, infrastructure, emergency response systems, and public trust, exposing the limitations of fragmented and nationally bounded healthcare systems and reinforcing the need to strengthen coordination capacity across providers, sectors, and governance levels [3–5, 25–27].

Evidence from crisis and conflict settings indicates that resilience depends not only on emergency preparedness but on the combined capacity of routine healthcare, public health systems, and coordinated governance structures, often operationalised through integrated models of care [3, 25, 26, 28]. In fragile and conflict-affected contexts, integrated responses, context-specific governance arrangements, and information-sharing mechanisms are key facilitators of system strengthening [26, 28].

These dynamics elevate health from a sectoral policy domain to a strategic component of national and regional security, creating pressure for structural reform [5, 27].

#### Reforms and incentive reconfiguration

In response to these pressures, health systems are undergoing reforms aimed at improving efficiency, sustainability, and crisis preparedness. The literature highlights a shift from volume-based models of care toward value-based healthcare, prioritising outcomes, coordination, and cost-effectiveness [12–14]. Central to this shift is the reconfiguration of financial and organisational incentives, as fee-for-service models discourage coordination across providers [29].

Emerging mechanisms, including bundled payments, shared savings, alternative payment models, and pay-for-performance, aim to align provider behaviour with integrated and multidisciplinary care delivery [21, 29, 30]. Reforms also involve strengthening governance, enhancing primary care, and expanding the use of data for decision-making and system learning [10]. However, implementation remains uneven, often constrained by limited infrastructure, particularly in information systems and financing mechanisms [14, 31].

Taken together, these developments suggest that health system integration functions as an important mechanism for operationalising value-based reforms, linking financial incentives with coordination across providers and sectors [13, 29].

#### Integration, digital transformation, and the emergence of digital health infostructure

The advancement of integrated healthcare is closely linked to digital health infostructure, understood here as the combined ecosystem of information systems, IT platforms, interoperability standards, data infrastructures, governance rules, and operational processes. Integrated care requires coordination across providers, sectors, and levels of care, supported by interoperable systems, governance alignment, and sustainable financing [17, 19, 21, 22, 32]. Digital transformation enables health information exchange, supports task redistribution, and may improve workforce efficiency by creating interoperable data environments [33–35]. Importantly, this infostructure extends beyond technical implementation to include legislative harmonisation, data governance, and process alignment [1, 24].

The expansion of telehealth, remote monitoring, electronic health records, and other digital health solutions during the COVID-19 pandemic illustrates how crisis conditions can accelerate digital integration [33, 35]. As integration and digitalisation advance, health systems increasingly generate and rely on large-scale data infrastructures. These enable population health management, resource allocation, and policy planning through data-driven approaches such as segmentation and predictive analytics [36]. Consequently, data becomes a central organising element of integrated health systems, underpinning both operational coordination and strategic decision-making.

#### Data interdependence and governance constraints

The expansion of integrated and digitally enabled systems increases interdependence between national health systems through shared data flows, coordinated responses, and global supply chains. However, governance mechanisms have often not evolved at the same pace. Evidence from the COVID-19 pandemic highlights persistent gaps in global health governance, including weak enforcement, fragmented institutional coordination, and limited accountability [37–42].

These limitations are reflected in initiatives such as COVAX, where cooperative models were undermined by vaccine nationalism and unequal resource distribution, illustrating tensions between collective needs and national priorities [37]. Similarly, international frameworks such as the International Health Regulations rely on voluntary compliance, limiting their effectiveness in ensuring coordinated responses [38]. Governance processes are further shaped by power asymmetries, with stronger actors exerting disproportionate influence [43]. At the same time, emerging initiatives such as the European Health Data Space aim to address some data-related governance constraints by harmonising legislation, processes, and data structures to facilitate access to health data for care and research [24].

As a result, increasing interdependence exposes structural weaknesses in governance systems, where coordination is necessary but not effectively ensured.

#### Cross-border cooperation under constraints

Under conditions of interdependence, cross-border cooperation becomes a functional necessity, as transnational health threats, including pandemic, One Health, conflict-related, and disinformation-driven risks, cannot be managed within national boundaries alone [38, 44–46]. However, cooperation remains constrained by divergent national interests, institutional fragmentation, unequal system capacities, and competing governance priorities.

Evidence indicates that cooperation depends on trust, governance compatibility, and aligned incentives rather than being systematically guaranteed [47]. Disparities in digital infrastructure, regulatory frameworks, and data governance further limit coordinated action, reinforcing inequalities and reducing the effectiveness of collective responses [48, 49].

As a result, cross-border cooperation emerges not as a stable or institutionalised process, but as a context-dependent and negotiated arrangement shaped by asymmetries in capacity, political priorities, and governance structures. This highlights the need for mechanisms capable of mediating between interdependence and constraint where formal governance frameworks remain insufficient. Public health diplomacy emerges as one such mechanism.

#### Emergence of public health diplomacy

While health diplomacy and global health diplomacy can be understood as umbrella terms and broadly encompass negotiation and governance processes addressing transnational health challenges, the literature has traditionally focused on multilateral interactions involving states, ministries, international organisations, donors, and non-governmental actors [6, 7, 50]. These approaches are primarily oriented towards the global policy environment for health and formal international coordination mechanisms, including how countries position themselves within global health governance [51–53]. However, more recent literature increasingly points towards operational, system-based forms of interaction emerging from within health systems themselves [8, 9]. These developments reflect a broader shift from predominantly state-centric diplomacy towards system-level interactions grounded in health system capacities, governance structures, and coordination mechanisms.

In this context, public health diplomacy expands participation beyond traditional diplomatic and governmental actors by involving public health professionals, local health agencies, academic institutions, professional networks, and community-oriented stakeholders [8, 54]. This broader level of participation emphasises communication, consensus-building, evidence translation, community engagement, and equity-oriented approaches to cross-border health challenges. At the same time, these processes remain inherently political, shaped by national security concerns, economic priorities, power asymmetries, and changing geopolitical conditions [6, 7, 55].

The growing importance of digital health further contributes to new forms of interaction, often described as digital health diplomacy, where data governance, technology access, and interoperability become central subjects of negotiation [48, 49]. In this framework, public health diplomacy can be understood as an operational layer of these system-level interactions, grounded in health system capacities, data infrastructures, and coordination mechanisms.

Overall, the findings were organised into a conceptual sequence linking six recurring thematic elements: systemic threats, integration-oriented reforms, digital health infostructure, data interdependence, governance constraints, and cross-border cooperation. This sequence was not identified as an explicit causal pathway in the included studies, but was derived through interpretation across the reviewed domains. It suggests that domestic health system transformation may generate coordination demands that extend beyond national systems, creating conditions in which public health diplomacy becomes relevant for managing transnational health risks, including pandemic preparedness and other cross-border challenges [56]. These conditions form the analytical foundation for the Health Diplomacy Spiral framework developed in the following section.

### Conceptual synthesis and framework development: the health diplomacy spiral

**FIGURE 2:**
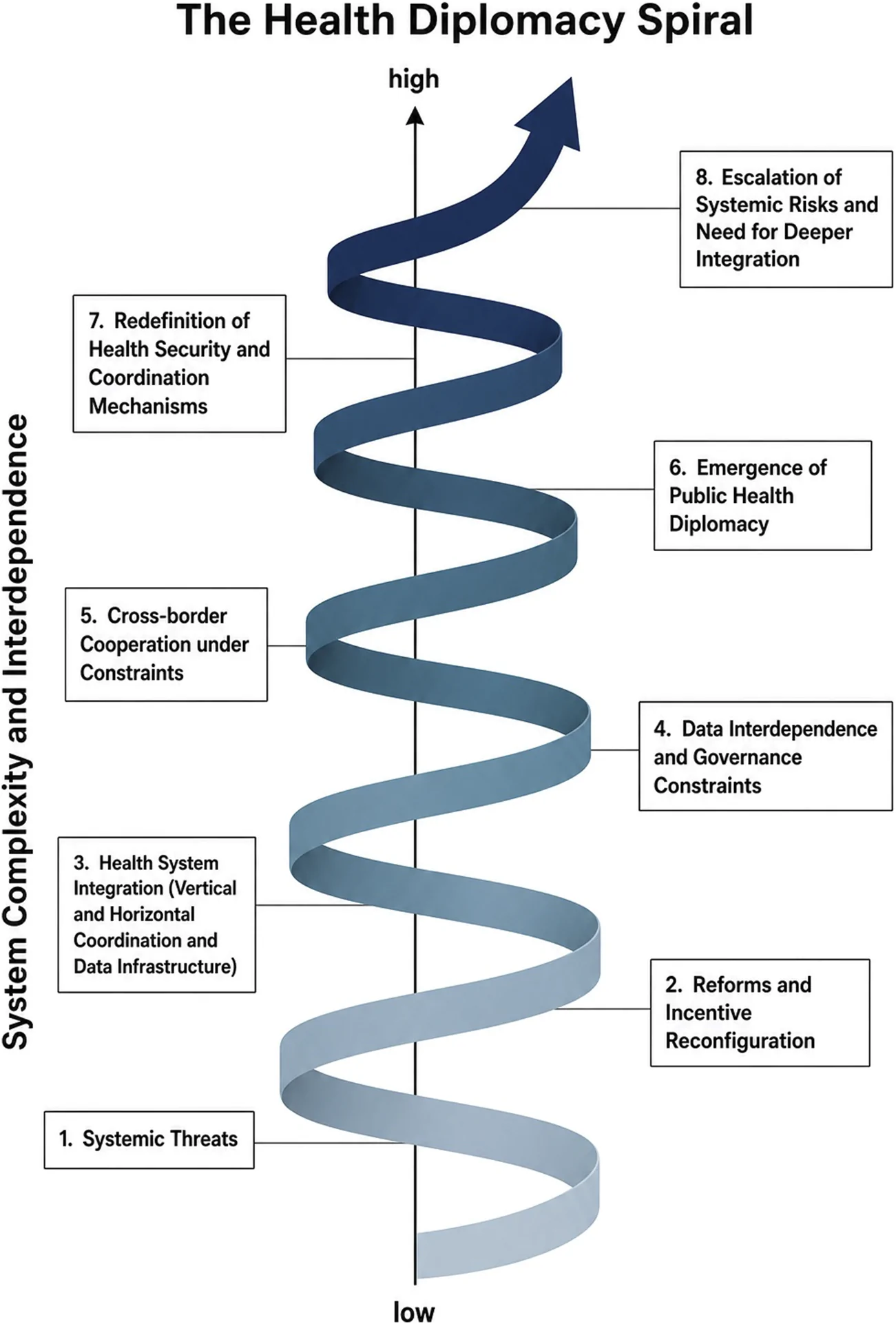
The Health Diplomacy Spiral: A conceptual framework linking health system threats and public health diplomacy, structured integrative literature review, Global 2015–2025.

The synthesis builds on the thematic sequence identified above. Although this relationship is not explicitly conceptualised in the literature, recurring cross-domain elements link systemic pressures, institutional reforms, integration processes, and cross-border coordination. These links informed development of the Health Diplomacy Spiral, a conceptual framework linking systemic threats, integration-oriented reforms, and cross-border governance dynamics (Figure 2). Rather than a linear or cyclical process, the model captures an iterative dynamic in which successive stages reshape health system structures and forms of international cooperation. As the spiral progresses, increasing interdependence, system complexity, and constraint elevate the role of coordination and negotiation, contributing to the emergence of public health diplomacy.

The spiral begins with systemic threats, including pandemics, geopolitical instability, and hybrid risks, which expose the limitations of fragmented and nationally bounded healthcare structures. These pressures increase uncertainty and coordination costs, elevating health from a sectoral issue to a matter of national and regional security. In response, governments pursue reforms aimed at resilience, efficiency, and preparedness, reconfiguring governance, financing, and incentive structures towards coordination, outcomes and system-wide performance. Health system integration thus emerges as a central strategy, combining vertical and horizontal coordination across providers, sectors, and levels of care.

As integration advances, it strengthens coordination capacity and enables the development of interoperable data infrastructures. Digital health solutions integrate clinical, administrative and population-level data into multi-institutional digital patient journeys, connecting islands of health-related information into a health data backbone, which becomes a central organising element of the system. This expansion also generates new forms of interdependence, as health systems increasingly rely on shared data flows for monitoring, forecasting and coordinated responses to transnational health threats.

This interdependence is accompanied by governance constraints. Differences in legal frameworks, privacy regulations and institutional arrangements create frictions in cross-border data use, while issues of sovereignty transform data into a politically sensitive asset. Health systems therefore operate under conditions of constrained interdependence, where the need for collaboration coexists with structural limitations on coordination.

Under these conditions, cross-border cooperation becomes necessary but contested. Effective collaboration depends on trust, aligned incentives, and compatible governance frameworks, but remains constrained by competing national interests. The coexistence of shared needs and institutional constraints generates demand for mechanisms capable of mediating and stabilising interactions across jurisdictions.

These conditions create the structural space for public health diplomacy, understood here not as an extension of foreign policy alone, but as an operational layer of system-level interactions responding to coordination challenges under constrained interdependence. In practice, this may include negotiation, standard-setting, institutional alignment, data-sharing arrangements, crisis coordination and the development of common regulatory approaches.

In contrast to predominantly state-centric forms of global health diplomacy, public health diplomacy expands participation to a broader range of actors embedded within health systems themselves, including public health professionals, local health agencies, academic institutions, professional networks, and community-oriented stakeholders. In this context, health systems function not only as objects of governance, but as operational platforms through which diplomatic interaction, coordination, and policy alignment are enacted.

Public health diplomacy, in turn, reshapes the understanding of health security by extending it beyond service delivery to include resilience, data governance, systemic preparedness, and coordination capacity. It contributes to the development of shared norms, policy coordination mechanisms, and institutionalised forms of cooperation, while enabling knowledge exchange, and policy learning across systems.

Finally, the spiral escalates as these processes reveal new forms of vulnerability. Increased reliance on data infrastructures and cross-border coordination introduces additional systemic risks, including data dependencies, interoperability constraints and exposure to hybrid threats. These vulnerabilities redefine the perception of systemic threats, generating renewed demands for resilience, integration and governance adaptation. The system thus re-enters the spiral at a higher level of complexity, reinforcing the dynamic interplay between health system transformation and public health diplomacy. This escalating dynamic underscore the iterative and open-ended nature of the Health Diplomacy Spiral.

## Discussion

This study examines the relationship between health system integration and the emergence of public health diplomacy. The findings identify an under-conceptualised pattern linking systemic pressures, integration-oriented reforms, and cross-border coordination. The Health Diplomacy Spiral addresses this gap by connecting domestic health system transformation with global health governance dynamics and capturing their iterative and escalating nature. Unlike a simple cycle, the spiral emphasises that each round of reform and coordination changes the conditions for the next: integration and digitalisation expand data flows and dependencies, governance constraints reveal new vulnerabilities. As a result, the system re-enters the following cycle at a higher level of complexity, interdependence, and diplomatic demand.

This contribution responds to a persistent divide in research and practice: health systems are analysed from a national perspective, while health diplomacy is examined through international institutions and multilateral processes. These perspectives often evolve separately, limiting understanding of how domestic transformations generate cross-border interdependencies and require new forms of coordination and negotiation.

Countries facing heightened systemic risks—including those on the eastern border of NATO—are simultaneously implementing rapid internal reforms while navigating external pressures requiring international coordination. The war in Ukraine and its implications for neighbouring health systems illustrate how geopolitical instability directly shapes both domestic reform agendas and cross-border health coordination needs [5]. In such settings, the distinction between domestic policy and international cooperation becomes blurred. The proposed framework addresses this by linking integration, digitalisation, and resilience-building with the emergence of public health diplomacy, providing a bridge between systemic threats and governance dynamics.

A second contribution concerns the conceptual positioning of public health diplomacy within the broader field of health diplomacy. The literature reflects overlap between global, public, and digital health diplomacy [8, 9]. Rather than redefining these terms, this study uses them to clarify the specific role of public health diplomacy in the proposed framework. Public health diplomacy is conceptualised as an operational layer of system-level interactions emerging from health systems themselves, grounded in coordination capacity, data infrastructures, and governance arrangements, and places greater emphasis on practice-oriented interactions arising from interdependencies and constraints than formal multilateral processes.

The findings also highlight directions for future research. Several examples discussed in this review illustrate how components of the Health Diplomacy Spiral may appear in practice: COVAX reflects tensions between collective health needs and national priorities, the European Health Data Space illustrates efforts to institutionalise cross-border data governance, and the war in Ukraine shows how geopolitical instability can reshape domestic preparedness and cross-border coordination needs [5, 24, 37]. These examples support the relevance of the framework, but they are not formal case studies. Future research should therefore apply the Health Diplomacy Spiral empirically to specific country or policy contexts, particularly rapidly transforming health systems and low- and middle-income countries where governance and interoperability constraints may be especially visible. Recent evidence from Ghana illustrates this potential, showing that digital health fragmentation reflected fragmented mandates, weak enforcement, donor-driven implementation, and constrained subnational capacity [57]. In addition, there remains a relative lack of structured research on health system transformation as a systemic process, especially in relation to healthcare financing reforms. Although value-based healthcare is widely discussed, evidence on how financial incentives, governance mechanisms, and integration processes interact in practice remains limited, representing an important gap given the role of funding in shaping system behaviour and coordination capacity. Future research should also examine how data governance frameworks and digital health infrastructures shape the scope and effectiveness of public health diplomacy, particularly as interoperability and data sovereignty become increasingly contested.

Beyond academic research, the Health Diplomacy Spiral may serve as a practical analytical tool for policymakers and health system managers navigating the intersection of domestic reforms and international coordination.

### Limitations

Several limitations should be acknowledged. The literature search was conducted using a single database, which may have limited the scope of identified studies, particularly in interdisciplinary fields such as governance and international relations. Although PubMed was considered appropriate for capturing health systems, public health, digital health, and health diplomacy literature, relevant work from political science, public administration, international relations, or broader global governance may have been missed. The search was also restricted to English-language publications and a defined time period, potentially excluding relevant earlier or non-English contributions. In addition, the study selection process did not involve independent dual screening, which may introduce selection bias despite the use of structured scoring criteria. Furthermore, the Health Diplomacy Spiral is a conceptual framework developed through literature synthesis and has not been empirically validated. Its applicability across different health system contexts remains to be tested.

Despite these limitations, the study contributes a conceptual perspective on the relationship between health system integration and public health diplomacy. By framing this relationship as an iterative and escalating process, the Health Diplomacy Spiral illustrates how increasing integration and interdependence create both opportunities and constraints for coordination. In this context, public health diplomacy is positioned as an intrinsic component of contemporary health system governance. The framework provides a basis for future empirical research and may inform further exploration of policy and governance implications.

### Conclusion

This study examined the interrelation between health system integration and the emergence of public health diplomacy through a integrative literature review and conceptual synthesis. The findings show that systemic threats, institutional reforms leading to system integration and cross-border coordination form an interconnected dynamic shaping contemporary health system.

To capture this dynamic, Health Diplomacy Spiral, a conceptual framework linking national health system transformations with evolving forms of international cooperation, was developed. The framework demonstrates how integration and digitalisation generate interdependence while exposing governance constraints that require coordination beyond national boundaries. In this context, public health diplomacy emerges not as an external policy function, but as an operational response to managing increasingly complex and interconnected systems. The iterative structure of the spiral further illustrates how each cycle of transformation and coordination generates new vulnerabilities, sustaining the dynamic between health system reform and international cooperation.

By bridging system-level reforms with global health governance, the study contributes to a more integrated understanding of health systems in an interdependent environment and provides a foundation for future research on the interaction between system design, data governance, and international coordination. The Health Diplomacy Spiral offers an analytical lens for understanding how health system transformation becomes embedded in broader geopolitical processes. As health systems grow more integrated and interdependent, frameworks that capture this complexity will be essential for both researchers and policymakers navigating an increasingly interconnected world.

## Data Availability

The original contributions presented in the study are included in the article and Supplementary Files 1 and 2. Further inquiries can be directed to the corresponding author.
