# Supplementary material for: The health diplomacy spiral: a conceptual framework linking health system integration and public health diplomacy
Source: Public Health Rev. 2026 Aug 25;47:1609977. doi: 10.3389/phrs.2026.1609977 (PMC13549955; doi:10.3389/phrs.2026.1609977)
Supplement: Supplementary file 1 [file Supplementaryfile1.docx]

Supplementary File 1. Characteristics of studies included in the structured literature review (n = 46)

| **Authors** | **Year** | **Title** | **Journal** | **Type of article** | **Literature domain** |
| --- | --- | --- | --- | --- | --- |
| McNeill D, Ottersen O P | 2015 | Global Governance for Health: How to motivate political change? | Public Health | Policy / practice analysis | Health diplomacy and global health governance |
| Kickbusch I, Reddy K S | 2015 | Global health governance - the next political revolution | Public Health | Policy / practice analysis | Health diplomacy and global health governance |
| Ruckert A, Labonté R, Lencucha R et al. | 2016 | Global health diplomacy: A critical review of the literature | Social Science and Medicine | Review | Health diplomacy and global health governance |
| Ramírez J, Valdivia L, Rivera E et al. | 2018 | Chile's role in global health diplomacy: A narrative literature review | Globalization and Health | Narrative review | Health diplomacy and global health governance |
| Asadi-Lari A, Teymourlouy A A, Maleki M et al. | 2021 | Opportunities and challenges of global health diplomacy for prevention and control of noncommunicable diseases: a systematic review | BMC Health Services Research | Systematic review | Health diplomacy and global health governance |
| Wæhler T A, Ingebrigtsen T | 2022 | Health risks, emergency preparedness and Norwegian-Russian cooperation on Svalbard. A systematic review | International Journal of Circumpolar Health | Systematic review | Health diplomacy and global health governance |
| Suzuki M, Webb D, Small R | 2022 | Competing Frames in Global Health Governance: An Analysis of Stakeholder Influence on the Political Declaration on Non-communicable Diseases | International Journal of Health Policy and Management | Review | Health diplomacy and global health governance |
| Godinho M A, Martins H, Al-Shorbaji et al. | 2022 | "Digital Health Diplomacy" in Global Digital Health? A call for critique and discourse | Journal of the American Medical Informatics Association | Review | Health diplomacy and global health governance |
| Kickbusch I, Liu A | 2022 | Global health diplomacy—reconstructing power and governance | The Lancet | Systematic review | Health diplomacy and global health governance |
| Mac-Seing M, Gidey M, Di Ruggiero E | 2023 | COVID-19-related global health governance and population health priorities for health equity in G20 countries: a scoping review | International Journal for Equity in Health | Scoping review | Health diplomacy and global health governance |
| Pushkaran A, Chattu V K, Narayanan P | 2023 | A critical analysis of COVAX alliance and corresponding global health governance and policy issues: A scoping review | BMJ Global Health | Scoping review | Health diplomacy and global health governance |
| Song P, Adeloye D, Acharya Y et al. | 2024 | Setting research priorities for global pandemic preparedness: An international consensus and comparison with ChatGPT’s output | Journal of Global Health | Consensus / priority-setting study | Health diplomacy and global health governance |
| Pitt S J, Gunn A | 2024 | The One Health Concept | British Journal of Biomedical Science | Policy / practice analysis | Health diplomacy and global health governance |
| Holzscheiter A, Franz C, Kickbusch I | 2024 | Germany's role in global health at a critical juncture | The Lancet | Review | Health diplomacy and global health governance |
| Lazarus J F, Pujol-Martinez C, Kopka C J et al. | 2024 | Implications from COVID-19 for future pandemic global health governance | Clinical Microbiology and Infection | Narrative review | Health diplomacy and global health governance |
| Wang C, Huang S, Lassi N et al. | 2025 | A decade of China’s health silk road: policy review for global health governance and SDG partnerships | Frontiers in Public Health | Review | Health diplomacy and global health governance |
| Abdel-Motaal K A, Chun S | 2025 | Governance in Crisis: A Mixed-Methods Analysis of Global Health Governance During COVID-19 | International Journal of Environmental Research and Public Health | Review | Health diplomacy and global health governance |
| Stepanskyi D, Castro K G | 2025 | From knowledge to action: the role of professional health associations on public health well-being in Ukraine | Globalization and Health | Review | Health diplomacy and global health governance |
| Abdel-Motaal K A, El Kheir-Mataria W A, Chun S | 2025 | Global Health Governance and the WHO Pandemic Agreement: A Scoping Review of Challenges and Analysis of Reforms | International Journal of Environmental Research and Public Health | Scoping review | Health diplomacy and global health governance |
| McKee M, Figueras J, Kalediene R et al. | 2025 | An Agenda for Public Health Diplomacy in an Age of Populism | Public Health Reviews | Original research / empirical study | Health diplomacy and global health governance |
| da Silva J B, Valdez M L, Sealey-Thomas R et al. | 2025 | Digital health diplomacy and universal health coverage | Bulletin of the World Health Organization | Policy / practice analysis | Health diplomacy and global health governance |
| Greer S L, Jarman H, Kulikoff R et al. | 2025 | The second Trump administration: A policy analysis of challenges and opportunities for European health policymakers | Health Policy | Policy analysis | Health diplomacy and global health governance |
| Peters L E R, Charnley G E C, Roberts S et al. | 2025 | Public health disinformation, conflict, and disease outbreaks: a global narrative integrative review to guide new directions for health diplomacy | Global Health Action | Narrative review | Health diplomacy and global health governance |
| Joshi A, Magana L, Tsai K H et al. | 2025 | Public health diplomacy: summary of the methods and outcome of the 1st University of Memphis School of Public Health Diplomacy Summit | Frontiers in Public Health | Consensus / priority-setting study | Health diplomacy and global health governance |
|  |  |  |  |  |  |
| Shinbane J S, Saxon L A | 2016 | Digital monitoring and care: Virtual medicine | Trends in Cardiovascular Medicine | Policy / practice analysis | Health system transformation |
| Raney L, Bergman D, Torous J et al. | 2017 | Digitally Driven Integrated Primary Care and Behavioural Health: How Technology Can Expand Access to Effective Treatment | Current Psychiatry Reports | Review | Health system transformation |
| Yan S, Kwan Y H, Tan C S et al. | 2018 | A systematic review of the clinical application of data-driven population segmentation analysis | BMC Medical Research Methodology | Systematic review | Health system transformation |
| Quaglio G, Figueras J, Mantoan D et al. | 2018 | Perspectives: An overview of future EU health systems. An insight into governance, primary care, data collection and citizens' participation | Journal of Public Health (United Kingdom) | Policy / practice analysis | Health system transformation |
| Heeringa J,Mutti A, Furukawa M F et al. | 2020 | Horizontal and vertical integration of health care providers: A framework for understanding various provider organizational structures | International Journal of Integrated Care | Narrative review | Health system transformation |
| Granholm F , Tin D, Ciottone G R | 2022 | Not war, not terrorism, the impact of hybrid warfare on emergency medicine | American Journal of Emergency Medicine | Policy / practice analysis | Health system transformation |
| Gratale D, Viveiros J, Boyer K | 2022 | Paediatric alternative payment models: Emerging elements | Current Opinion in Pediatrics | Review | Health system transformation |
| Sum G, Lau L K, Jabbar K A et al. | 2023 | The World Health Organization (WHO) Integrated Care for Older People (ICOPE) Framework: A Narrative Review on Its Adoption Worldwide and Lessons Learnt | International Journal of Environmental Research and Public Health | Narrative review | Health system transformation |
| Epstein A, Lim R, Johannigman J et al. | 2023 | Putting Medical Boots on the Ground: Lessons from the War in Ukraine and Applications for Future Conflict with Near-Peer Adversaries | Journal of the American College of Surgeons | Review | Health system transformation |
| Lim A W K, Tan C, Yap C H J | 2024 | Measuring the Maturity of Integrated Care in Singapore with the SCIROCCO Exchange Tool | International Journal of Integrated Care | Consensus / priority-setting study | Health system transformation |
| Westerink H J, Steinmann G, Koomans M et al. | 2024 | Value-based healthcare implementation in the Netherlands: a quantitative analysis of multidisciplinary team performance | BMC Health Services Research | Case study / country analysis | Health system transformation |
| Chen W T, LimS Y, How S et al. | 2024 | Communities of Care Approach: Developing a Place-based Model of Care and Building Partnerships in the Communities in Central Singapore | International Journal of Integrated Care | Case study / country analysis | Health system transformation |
| Fernández-Salido M, Alhambra-Borrás T, Casanova G et al. | 2024 | Value-Based Healthcare Delivery: A Scoping Review | International Journal of Environmental Research and Public Health | Scoping review | Health system transformation |
| Bogale B, Scambler S, Khairuddin A N M et al. | 2024 | Health system strengthening in fragile and conflict-affected states: A review of systematic reviews | PLoS ONE | Systematic review | Health system transformation |
| van Hoorn E S, Ye L, van Leeuwen N et al. | 2024 | Value-Based Integrated Care: A Systematic Literature Review | International Journal of Health Policy and Management | Systematic review | Health system transformation |
| Döbrössy B, Girasek E, Győrffy Z | 2024 | The Adaptation of Digital Health Solutions During the COVID-19 Pandemic in Hungary: A Scoping Review | International Journal of Health Policy and Management | Scoping review | Health system transformation |
| Yordanov D, Oxholm A S, Prætorius T et al. | 2024 | Financial incentives for integrated care: A scoping review and lessons for evidence-based design | Health Policy | Scoping review | Health system transformation |
| Khorram-Manesh A, Burkle F M | 2024 | The Role of Health Care and Health Security in War, Disaster & Crisis Preparedness | Disaster Medicine and Public Health Preparedness | Original research / empirical study | Health system transformation |
| Mercogliano M, Spatari G, Noviello C et al. | 2025 | Building evidences in Public Health Emergency Preparedness (“BePHEP” Project)—a systematic review | International Journal for Equity in Health | Systematic review | Health system transformation |
| Ugwu C N, Ugwu O P C, Alum E U et al. | 2025 | Sustainable development goals (SDGs) and resilient healthcare systems: Addressing medicine and public health challenges in conflict zones | Medicine | Review | Health system transformation |
| Goralnick E, Holcomb J B, Elster E A | 2025 | Preparing the Civilian Health Care System for Wartime: A National Imperative for Military-Civilian Integration | JAMA | Policy / practice analysis | Health system transformation |
| Grozdanova A, Mihajloska E, Naumovska Z et al. | 2025 | Toward value in healthcare: opportunities and challenges for implementing Value-Based Healthcare in North Macedonia | Frontiers in Public Health | Review | Health system transformation |
