# Supplementary material for: The health diplomacy spiral: a conceptual framework linking health system integration and public health diplomacy
Source: Public Health Rev. 2026 Aug 25;47:1609977. doi: 10.3389/phrs.2026.1609977 (PMC13549955; doi:10.3389/phrs.2026.1609977)
Supplement: Supplementary file 2 [file Supplementaryfile2.docx]

**Supplementary File 2. PubMed Search Strategy and Abstract Screening Scoring Framework**

This supplementary file provides the PubMed search strategy and the abstract screening scoring framework used in the structured integrative literature review.

**1. PubMed search strategy**

The literature search was conducted in PubMed and covered publications from January 2015 to December 2025. The search was limited to English-language publications. The search strategy was organised into two complementary blocks: health system transformation and health diplomacy/global health governance.

The first block focused on health system transformation, including integrated care, digital health transformation, data governance, health system resilience and preparedness, value-based healthcare, and payment mechanisms. The second block focused on health diplomacy, public health diplomacy, global health diplomacy, and global health governance.

Table S2.1. PubMed search blocks used in the structured integrative literature review

| **Search block** | **Domain** | **Search string** |
| --- | --- | --- |
| Block 1 | Health system transformation | ("integrated care"[Title/Abstract] OR "integrated healthcare"[Title/Abstract] OR "Delivery of Health Care, Integrated"[MeSH]) AND ("digital health"[Title/Abstract] OR "digital transformation"[Title/Abstract] OR "health data"[Title/Abstract] OR "data governance"[Title/Abstract] OR "digital health governance"[Title/Abstract] OR "health system resilience"[Title/Abstract] OR "health system preparedness"[Title/Abstract] OR "healthcare system resilience"[Title/Abstract] OR "crisis preparedness"[Title/Abstract] OR "emergency preparedness"[Title/Abstract] OR "Disaster Planning"[MeSH] OR "Emergency Preparedness"[MeSH] OR "value-based healthcare"[Title/Abstract] OR "value-based care"[Title/Abstract] OR "pay for performance"[Title/Abstract] OR "Pay for Performance"[MeSH] OR "Value-Based Purchasing"[MeSH]) |
| Block 2 | Health diplomacy and global health governance | "Health diplomacy"[Title/Abstract] OR "global health diplomacy"[Title/Abstract] OR "public health diplomacy"[Title/Abstract] OR "global health governance"[Title/Abstract] |

**2. Abstract screening scoring framework**

A structured scoring framework was applied during title and abstract screening. The purpose of the scoring framework was to support transparent and consistent selection of studies relevant to the conceptual synthesis. Each study could receive a maximum score of six points.

Five screening criteria were scored from 0 to 1 point. One additional point was awarded for recency, defined as publication between 2020 and 2025.

Studies scoring four points or higher were retained for full-text review. The threshold of four points was selected to retain studies with at least moderate relevance across the screening criteria, while excluding studies that were primarily clinical, disease-specific, patient-level, single-provider, technical, or peripheral to the conceptual synthesis.

Recency was included as an additional point because the review aimed to capture recent developments in health system transformation, digital health, resilience, governance, and diplomacy. However, recency did not override conceptual relevance. Older studies could still be included if they were conceptually important and met the predefined threshold.

Table S2.2. Abstract screening scoring framework

| **No.** | **Screening criterion** | **What was assessed** | **Score** |
| --- | --- | --- | --- |
| 1 | System-level relevance | Whether the study addressed health systems, governance, policy, reform, or national/regional-level processes. | 0 or 1 |
| 2 | Alignment with core themes | Whether the study related to integration, digital health, resilience, value-based care, health diplomacy, or global health governance. | 0 or 1 |
| 3 | Contribution to the conceptual chain | Whether the study supported relationships relevant to the conceptual synthesis, such as integration and data, digitalization and coordination, or resilience and system transformation. | 0 or 1 |
| 4 | Analytical or conceptual value | Whether the study was a review, framework, policy analysis, evaluation, or other analytical publication rather than a purely descriptive or technical report. | 0 or 1 |
| 5 | Relevance for manuscript synthesis | Whether the study was likely to contribute to the Introduction, Results, Discussion, or conceptual framework. | 0 or 1 |
| 6 | Recency bonus | One additional point was awarded to publications from 2020 to 2025. | 0 or 1 |

**3. Examples of screening decisions**

Table S2.3. Examples of study evaluation during screening

| **Example type** | **Typical scoring rationale** | **Decision** |
| --- | --- | --- |
| Review or framework addressing integrated care, value-based healthcare, or system-level reform | High system-level relevance, direct alignment with core themes, and clear contribution to the conceptual synthesis. | Included |
| Policy or conceptual article addressing health diplomacy, global health governance, or public health diplomacy | High relevance to diplomacy and governance domains and clear contribution to the conceptual synthesis. | Included |
| Article focused mainly on a clinical intervention, disease-specific pathway, or individual provider setting | Limited system-level relevance and weak contribution to the conceptual synthesis. | Excluded |
| Technical digital health article without governance, interoperability, integration, or system transformation relevance | May mention digital health but lacks relevance to the conceptual framework. | Excluded |
| Descriptive article with limited analytical or conceptual contribution | Partial thematic relevance but limited contribution to framework development. | Excluded unless total score met the threshold |
